# *N*,*N*′-Bis[(*E*)-2,4,6-trimethyl­benzyl­idene]ethane-1,2-diamine

**DOI:** 10.1107/S1600536811029424

**Published:** 2011-07-30

**Authors:** Nonzaliseko Yumata, Thomas Gerber, Eric Hosten, Richard Betz

**Affiliations:** a Nelson Mandela Metropolitan University, Summerstrand Campus, Department of Chemistry, University Way, Summerstrand, PO Box 77000, Port Elizabeth 6031, South Africa

## Abstract

The title compound, C_22_H_28_N_2_, which is a double imine derived from ethane-1,2-diamine and mesityl aldehyde, has crystallographic inversion symmetry, with both C=N bonds *E* configured. The dihedral angle between the mesityl ring system and the imide functional group is 23.89 (17)°.

## Related literature

For background to applications of chelate complexes, see: Gade (1998 ▶). For the crystal structure of a palladium coord­ination compound involving the title compound as a ligand, see: Arici *et al.* (2006 ▶).
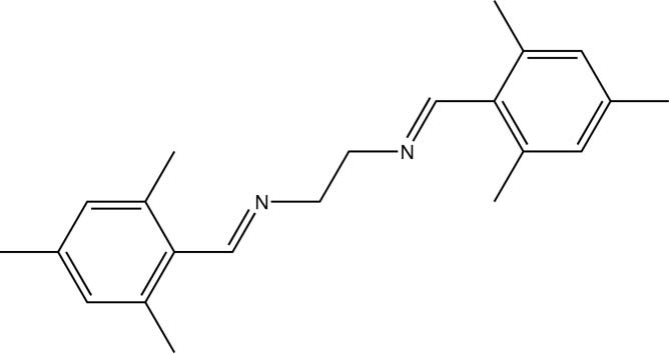


## Experimental

### 

#### Crystal data

C_22_H_28_N_2_
*M* *_r_* = 320.46
Monoclinic, 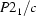

*a* = 11.1346 (5) Å
*b* = 5.2082 (2) Å
*c* = 15.9958 (7) Å
β = 93.154 (2)°
*V* = 926.21 (7) Å^3^
*Z* = 2
Mo *K*α radiation
μ = 0.07 mm^−1^
*T* = 200 K
0.21 × 0.09 × 0.07 mm

#### Data collection

Bruker APEXII CCD diffractometer
8541 measured reflections
2298 independent reflections
1205 reflections with *I* > 2σ(*I*)
*R*_int_ = 0.066

#### Refinement

*R*[*F* ^2^ > 2σ(*F* ^2^)] = 0.047
*wR*(*F* ^2^) = 0.113
*S* = 0.89
2298 reflections
112 parameters
H-atom parameters constrained
Δρ_max_ = 0.21 e Å^−3^
Δρ_min_ = −0.18 e Å^−3^

### 

Data collection: *APEX2* (Bruker, 2010 ▶); cell refinement: *SAINT* (Bruker, 2010 ▶); data reduction: *SAINT*; program(s) used to solve structure: *SHELXS97* (Sheldrick, 2008 ▶); program(s) used to refine structure: *SHELXL97* (Sheldrick, 2008 ▶); molecular graphics: *ORTEP-3* (Farrugia, 1997 ▶) and *Mercury* (Macrae *et al.*, 2008 ▶); software used to prepare material for publication: *SHELXL97* and *PLATON* (Spek, 2009 ▶).

## Supplementary Material

Crystal structure: contains datablock(s) I, global. DOI: 10.1107/S1600536811029424/zs2130sup1.cif

Supplementary material file. DOI: 10.1107/S1600536811029424/zs2130Isup2.cdx

Structure factors: contains datablock(s) I. DOI: 10.1107/S1600536811029424/zs2130Isup3.hkl

Supplementary material file. DOI: 10.1107/S1600536811029424/zs2130Isup4.cml

Additional supplementary materials:  crystallographic information; 3D view; checkCIF report

## Figures and Tables

**Fig. 1.**
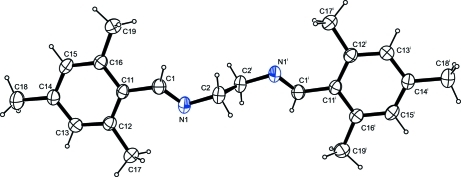
The molecular structure of the title compound, with atom labels and anisotropic displacement ellipsoids (drawn at the 50% probability level). For symmetry code (i): -x + 1, -y, -z.

**Fig. 2.**
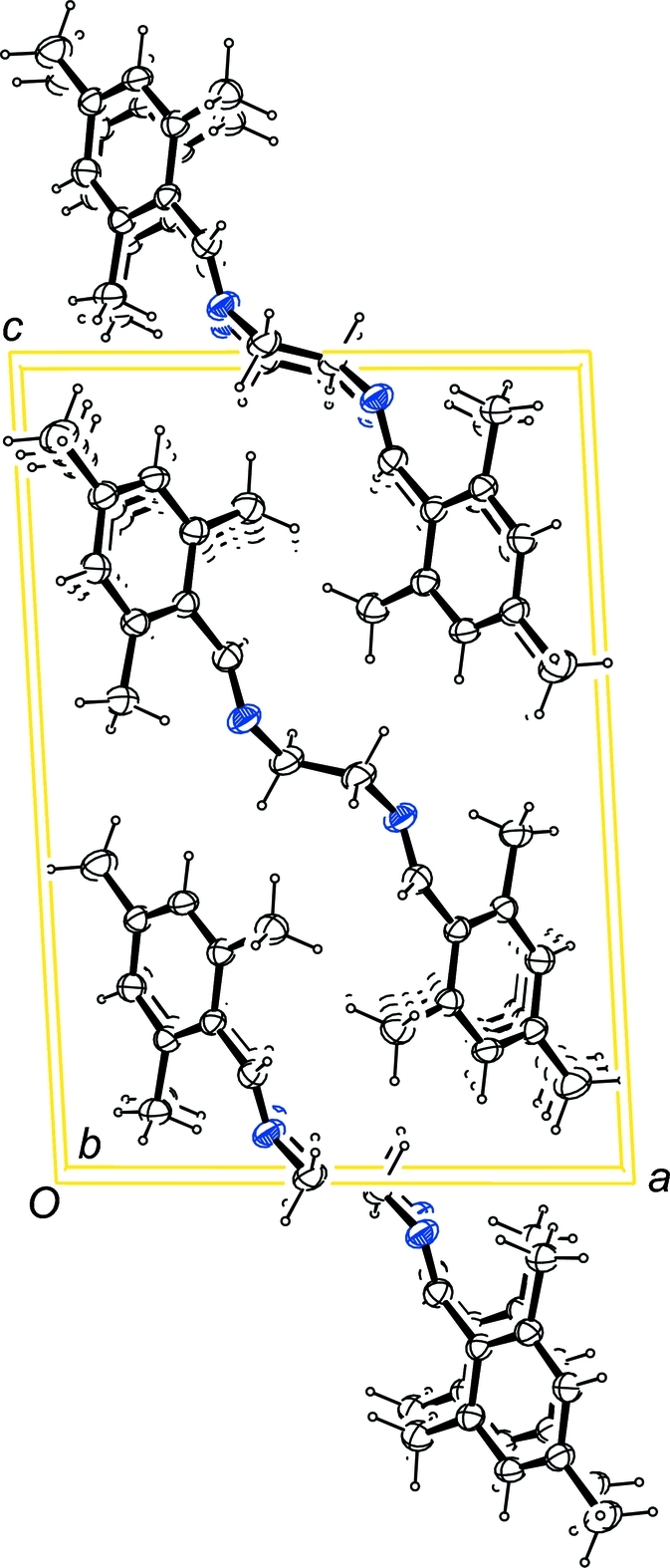
Molecular packing of the title compound, viewed along [0 1 0] (anisotropic displacement ellipsoids drawn at the 50% probability level).

## supplementary crystallographic information

### Comment

Chelate ligands have found widespread use in coordination chemistry due to the enhanced thermodynamic stability of resultant metal complexes in relation to those compounds involving comparable monodentate ligands exclusively (Gade, 1998). In our continuing efforts in elucidating the rules governing the formation of metal complexes with nitrogen-containing chelate ligands, we determined the structure of the title compound, C_22_H_28_N_2_, to allow comparative studies on designed coordination compounds. Structural information on a palladium(II) complex featuring the title compound as a ligand is found in the literature (Arici *et al.*, 2006).

The title compound has crystallographic inversion symmetry, the asymmetric unit comprising half a molecule, with both double-bonds (*E*)-configured (Fig. 1). Mesomeric interaction between the aromatic systems and the imine groups is hampered by the presence of the bulky methyl groups in the mesityl moiety which is evident in the out-of-plane orientation of the functional group of the Schiff-base. The dihedral angle between the least-squares planes defined by the carbon atoms of the mesityl group and those of the imine functional group (C—N═C) is 23.89 (17)°.

In the crystal structure, no significant intermolecular interactions are present (Fig. 2). The shortest inter-centroid distance between two aromatic ring systems was found to be 5.6101 (9) Å.

### Experimental

The title compound was prepared from the reaction of ethylenediamine (0.017 mol) and 2,4,6-trimethylbenzaldehyde (0.034 mol) at room temperature for two hours. The colourless title compound was filtered and recrystallized from methanol.

### Refinement

Carbon-bound H atoms were placed in calculated positions (C—H = 0.95–0.99 Å) and were included in the refinement in the riding model approximation, with *U_iso_*(H) set to 1.2*U*_eq_(aromatic and methylene C) or 1.5*U*_eq_(methyl C). The H atoms of the methyl groups were allowed to rotate.

### Crystal data

**Table:**

C_22_H_28_N_2_	*F*(000) = 348
*M**_r_* = 320.46	*D*_x_ = 1.149 Mg m^−^^3^
Monoclinic, *P*2_1_/*c*	Mo *K*α radiation, λ = 0.71073 Å
Hall symbol: -P 2ybc	Cell parameters from 1409 reflections
*a* = 11.1346 (5) Å	θ = 2.6–25.2°
*b* = 5.2082 (2) Å	µ = 0.07 mm^−^^1^
*c* = 15.9958 (7) Å	*T* = 200 K
β = 93.154 (2)°	Rod, colourless
*V* = 926.21 (7) Å^3^	0.21 × 0.09 × 0.07 mm
*Z* = 2	

### Data collection

**Table:**

Bruker APEXII CCD diffractometer	1205 reflections with *I* > 2σ(*I*)
Radiation source: fine-focus sealed tube	*R*_int_ = 0.066
graphite	θ_max_ = 28.3°, θ_min_ = 1.8°
φ and ω scans	*h* = −14→14
8541 measured reflections	*k* = −6→6
2298 independent reflections	*l* = −21→21

### Refinement

**Table:**

Refinement on *F*^2^	Primary atom site location: structure-invariant direct methods
Least-squares matrix: full	Secondary atom site location: difference Fourier map
*R*[*F*^2^ > 2σ(*F*^2^)] = 0.047	Hydrogen site location: inferred from neighbouring sites
*wR*(*F*^2^) = 0.113	H-atom parameters constrained
*S* = 0.89	*w* = 1/[σ^2^(*F*_o_^2^) + (0.0511*P*)^2^] where *P* = (*F*_o_^2^ + 2*F*_c_^2^)/3
2298 reflections	(Δ/σ)_max_ < 0.001
112 parameters	Δρ_max_ = 0.21 e Å^−^^3^
0 restraints	Δρ_min_ = −0.18 e Å^−^^3^

### Fractional atomic coordinates and isotropic or equivalent isotropic displacement parameters (Å^2^)

**Table:**

	*x*	*y*	*z*	*U*_iso_*/*U*_eq_	
N1	0.37236 (13)	0.1259 (3)	0.05808 (8)	0.0384 (4)	
C1	0.34978 (14)	0.0574 (3)	0.13110 (10)	0.0315 (4)	
H1	0.3771	−0.1078	0.1486	0.038*	
C2	0.43958 (14)	−0.0558 (3)	0.00940 (10)	0.0400 (5)	
H2A	0.3934	−0.0954	−0.0437	0.048*	
H2B	0.4514	−0.2177	0.0412	0.048*	
C11	0.28497 (13)	0.2094 (3)	0.19186 (9)	0.0275 (4)	
C12	0.20161 (14)	0.4033 (3)	0.16864 (9)	0.0297 (4)	
C13	0.14202 (14)	0.5283 (3)	0.23055 (9)	0.0340 (4)	
H13	0.0859	0.6593	0.2147	0.041*	
C14	0.16123 (14)	0.4694 (3)	0.31499 (10)	0.0322 (4)	
C15	0.24383 (14)	0.2802 (3)	0.33654 (9)	0.0320 (4)	
H15	0.2584	0.2385	0.3940	0.038*	
C16	0.30645 (14)	0.1486 (3)	0.27741 (9)	0.0290 (4)	
C17	0.17222 (15)	0.4778 (3)	0.07837 (10)	0.0398 (5)	
H17A	0.1050	0.5996	0.0756	0.060*	
H17B	0.1498	0.3240	0.0458	0.060*	
H17C	0.2428	0.5579	0.0552	0.060*	
C18	0.09542 (17)	0.6100 (3)	0.38071 (11)	0.0487 (5)	
H18A	0.1299	0.5635	0.4364	0.073*	
H18B	0.0101	0.5625	0.3762	0.073*	
H18C	0.1034	0.7955	0.3723	0.073*	
C19	0.39721 (15)	−0.0503 (3)	0.30684 (10)	0.0394 (4)	
H19A	0.4074	−0.0454	0.3681	0.059*	
H19B	0.4744	−0.0142	0.2827	0.059*	
H19C	0.3689	−0.2209	0.2889	0.059*	

### Atomic displacement parameters (Å^2^)

**Table:**

	*U*^11^	*U*^22^	*U*^33^	*U*^12^	*U*^13^	*U*^23^
N1	0.0414 (8)	0.0446 (9)	0.0303 (8)	0.0052 (7)	0.0114 (6)	−0.0025 (7)
C1	0.0324 (9)	0.0305 (9)	0.0319 (9)	−0.0017 (7)	0.0039 (7)	−0.0024 (7)
C2	0.0443 (11)	0.0457 (11)	0.0308 (9)	0.0052 (9)	0.0099 (8)	−0.0067 (8)
C11	0.0271 (8)	0.0303 (9)	0.0255 (8)	−0.0059 (7)	0.0049 (7)	−0.0009 (7)
C12	0.0297 (9)	0.0327 (9)	0.0267 (9)	−0.0040 (7)	0.0023 (7)	0.0001 (7)
C13	0.0332 (9)	0.0365 (10)	0.0321 (9)	0.0045 (8)	0.0014 (7)	−0.0005 (8)
C14	0.0330 (9)	0.0355 (10)	0.0286 (9)	−0.0016 (8)	0.0058 (7)	−0.0048 (7)
C15	0.0372 (10)	0.0367 (10)	0.0224 (8)	−0.0068 (8)	0.0035 (7)	0.0017 (7)
C16	0.0309 (9)	0.0283 (9)	0.0281 (9)	−0.0051 (7)	0.0037 (7)	0.0022 (7)
C17	0.0418 (10)	0.0490 (11)	0.0288 (9)	0.0045 (9)	0.0028 (8)	0.0044 (8)
C18	0.0547 (12)	0.0546 (13)	0.0377 (11)	0.0095 (10)	0.0118 (9)	−0.0082 (9)
C19	0.0425 (10)	0.0408 (10)	0.0349 (10)	0.0029 (9)	0.0024 (8)	0.0065 (8)

### Geometric parameters (Å, °)

**Table:**

N1—C1	1.2597 (19)	C14—C18	1.504 (2)
N1—C2	1.458 (2)	C15—C16	1.387 (2)
C1—C11	1.473 (2)	C15—H15	0.9500
C1—H1	0.9500	C16—C19	1.505 (2)
C2—C2^i^	1.511 (3)	C17—H17A	0.9800
C2—H2A	0.9900	C17—H17B	0.9800
C2—H2B	0.9900	C17—H17C	0.9800
C11—C12	1.408 (2)	C18—H18A	0.9800
C11—C16	1.412 (2)	C18—H18B	0.9800
C12—C13	1.385 (2)	C18—H18C	0.9800
C12—C17	1.513 (2)	C19—H19A	0.9800
C13—C14	1.390 (2)	C19—H19B	0.9800
C13—H13	0.9500	C19—H19C	0.9800
C14—C15	1.379 (2)		
			
C1—N1—C2	116.49 (14)	C14—C15—H15	118.8
N1—C1—C11	126.14 (15)	C16—C15—H15	118.8
N1—C1—H1	116.9	C15—C16—C11	119.02 (15)
C11—C1—H1	116.9	C15—C16—C19	118.77 (15)
N1—C2—C2^i^	110.22 (17)	C11—C16—C19	122.19 (14)
N1—C2—H2A	109.6	C12—C17—H17A	109.5
C2^i^—C2—H2A	109.6	C12—C17—H17B	109.5
N1—C2—H2B	109.6	H17A—C17—H17B	109.5
C2^i^—C2—H2B	109.6	C12—C17—H17C	109.5
H2A—C2—H2B	108.1	H17A—C17—H17C	109.5
C12—C11—C16	119.39 (14)	H17B—C17—H17C	109.5
C12—C11—C1	123.46 (14)	C14—C18—H18A	109.5
C16—C11—C1	117.12 (14)	C14—C18—H18B	109.5
C13—C12—C11	118.92 (14)	H18A—C18—H18B	109.5
C13—C12—C17	118.36 (15)	C14—C18—H18C	109.5
C11—C12—C17	122.71 (14)	H18A—C18—H18C	109.5
C12—C13—C14	122.47 (16)	H18B—C18—H18C	109.5
C12—C13—H13	118.8	C16—C19—H19A	109.5
C14—C13—H13	118.8	C16—C19—H19B	109.5
C15—C14—C13	117.75 (14)	H19A—C19—H19B	109.5
C15—C14—C18	121.14 (15)	C16—C19—H19C	109.5
C13—C14—C18	121.11 (16)	H19A—C19—H19C	109.5
C14—C15—C16	122.43 (15)	H19B—C19—H19C	109.5
			
C2—N1—C1—C11	179.13 (14)	C12—C13—C14—C15	0.5 (2)
C1—N1—C2—C2^i^	−115.7 (2)	C12—C13—C14—C18	179.66 (16)
N1—C1—C11—C12	25.0 (2)	C13—C14—C15—C16	−0.4 (2)
N1—C1—C11—C16	−156.94 (15)	C18—C14—C15—C16	−179.55 (16)
C16—C11—C12—C13	−0.5 (2)	C14—C15—C16—C11	−0.1 (2)
C1—C11—C12—C13	177.53 (15)	C14—C15—C16—C19	178.47 (15)
C16—C11—C12—C17	−179.17 (14)	C12—C11—C16—C15	0.6 (2)
C1—C11—C12—C17	−1.2 (2)	C1—C11—C16—C15	−177.54 (14)
C11—C12—C13—C14	−0.1 (2)	C12—C11—C16—C19	−177.96 (14)
C17—C12—C13—C14	178.66 (14)	C1—C11—C16—C19	3.9 (2)

Symmetry codes: (i) −*x*+1, −*y*, −*z*.
